# Increased exposure to sodium during pregnancy and lactation changes basal and induced behavioral and neuroendocrine responses in adult male offspring

**DOI:** 10.14814/phy2.13210

**Published:** 2017-03-29

**Authors:** Marcia S. Silva, Fabiana Lúcio‐Oliveira, Andre Souza Mecawi, Lucas F. Almeida, Silvia G. Ruginsk, Michael P. Greenwood, Mingkwan Greenwood, Laura Vivas, Lucila L. K. Elias, David Murphy, José Antunes‐Rodrigues

**Affiliations:** ^1^ Department of PhysiologySchool of Medicine of Ribeirao PretoUniversity of Sao PauloRibeirao PretoSão PauloBrazil; ^2^ Department of PhysiologyFaculty of Medicine, University of MalayaKuala LumpurMalaysia; ^3^ Department of Physiological SciencesInstitute of BiologyFederal Rural University of Rio de JaneiroRio de JaneiroSeropedicaBrazil; ^4^ Departament of Physiological SciencesBiomedical Sciences InstituteFederal University of AlfenasAlfenasMinas GeraisBrazil; ^5^ School of Clinical SciencesUniversity of BristolBristolUnited Kingdom; ^6^ Instituto de Investigación Médica Mercedes y Martín Ferreyra (INIMEC‐CONICET)Universidad Nacional de CórdobaCórdobaArgentina; ^7^ Facultad de Ciencias ExactasFísicas y NaturalesUniversidad Nacional de CórdobaCórdobaArgentina

**Keywords:** Perinatal programming, sodium preference ratio, thirst, vasopressin

## Abstract

Excessive sodium (Na^+^) intake in modern society has been associated with several chronic disorders such as hypertension. Several studies suggest that early life events can program physiological systems and lead to functional changes in adulthood. Therefore, we investigated behavioral and neuroendocrine responses under basal conditions and after 48 h of water deprivation in adult (60‐day‐old Wistar rats) male, Wistar rats originating from dams were offered only water or 0.15 mol/L NaCl during pregnancy and lactation. Early life salt exposure induced kidney damage, as shown by a higher number of ED‐1 positive cells (macrophages/monocytes), increased daily urinary volume and Na^+^ excretion, blunted basal water intake and plasma oxytocin levels, and increased plasma corticosterone secretion. When challenged with water deprivation, animals exposed to 0.15 mol/L NaCl during early life showed impaired water intake, reduced salt preference ratio, and vasopressin (AVP) secretion. In summary, our data demonstrate that the perinatal exposure to excessive Na^+^ intake can induce kidney injury in adult offspring and significantly affect the key mechanisms regulating water balance, fluid intake, and AVP release in response to water deprivation. Collectively, these novel results highlight the impact of perinatal programming on the homeostatic mechanisms regulating fluid and electrolyte balance during exposure to an environmental stress (i.e. dehydration) in later life.

## Introduction

Sodium (Na^+^) supplementation is common in the modern diet, mainly for the purposes of conserving and accentuating food taste (Gray et al. 2015; World Health Organization 2015). Na^+^ is the most abundant electrolyte found in extracellular fluid (ECF), where it determines the osmolality and volume of this compartment, and directly contributes to arterial pressure maintenance (Mecawi et al. 2015). Therefore, excessive dietary Na^+^ consumption has emerged as a major health problem in modern society, being associated with the development of several life‐threatening disorders, such as hypertension, chronic kidney disease, and stroke (Schmidt et al. 2011; Gray et al. 2015; World Health Organization 2015).

Epidemiological and experimental studies showed that the developmental disturbances can result in long‐life changes in several physiological systems (El‐Haddad et al. 2004; Koleganova et al. 2011; Lv et al. 2014; Maccari et al. 2014). A concept first introduced by Barker et al. (1986), developmental programming is a phenomenon that allows the fetus to respond to intrauterine or young life environment challenges in order to fine‐tune its homeostatic systems, shaping tissue structure and function, resulting in changes in the physiologic responses to environmental challenges in adulthood (Swanson et al. 2009; Babenko et al. 2015). Developmental programming may offer new perspectives on the investigation of modifiable risk factors in animal and human physiological and pathophysiological contexts. For instance, the excessive Na^+^ intake is not only associated with the development and maintenance of chronic diseases in adults (Brown et al. 2009), but also could affect the development/function of the osmoregulatory systems that control thirst, sodium appetite, and neuroendocrine release of osmoregulatory hormones (da Silva et al. 2003; Coelho et al. 2006; Lopes et al. 2008; Mecawi et al. 2015). Argüelles et al. (1996) suggested that high amounts of salt in the uterine environment could alter the sensitivity of the cardiovascular responses to pressor substances, such as angiotensin II (ANG II). Furthermore, adult offspring of dams fed with a high‐salt diet during pregnancy had increased blood pressure, and increased renal levels of ANG II (Leandro et al. 2008).

Coimbra et al. (2012) reported that high‐Na^+^ diet during pregnancy and lactation produced disturbances in offspring leading to functional (higher blood pressure) and structural alterations (lower number of glomeruli and tubulointerstitial lesions) that persisted in adult life in rats. Furthermore, studies performed by Macchione et al. (2012) demonstrated that the voluntary hypertonic Na^+^ consumption during the ontogenic period could induce, in the adult offspring, a permanent decrease in water and sodium intakes, as well as enhance vasopressinergic neuronal activity in the hypothalamus. These results were explained by the sensitization of lamina terminalis‐hypothalamus pathway induced by increased perinatal Na^+^ availability, allowing the imprinted offspring to have a larger anticipatory outcome in response to furosemide treatment, drinking less water in relation to hypertonic Na^+^ solution (increased osmolar intake), and reabsorbing more water (because of increased vasopressinergic activation) (Macchione et al. 2012).

We hypothesized that an adverse perinatal and postnatal environmental can induce permanent adaptive changes in the developing fetus, which may promote short‐term survival but may increase vulnerability to changes in several systems, like the system of hydromineral and neuroendocrine balance. Therefore, in the present study, we investigated the effects of maternal and offspring exposure to an isotonic (0.15 mol/L) NaCl solution during pregnancy and lactation on the behavioral and neuroendocrine responses in the adult male offspring, either under basal conditions or in response to a 48‐h water deprivation challenge.

## Materials and Methods

### Animals

Adult (60 days) female (180–200 g) and male (260–300 g) Wistar rats, obtained from the Animal Facility of the Campus of Ribeirao Preto, University of Sao Paulo, Brazil, were housed under controlled conditions of temperature (22–23°C) with a fixed light–dark cycle (lights off from 7:00 P.M. to 7:00 A.M.), with free access to standard food pellets (@Quimtia, Buenos Aires, Argentina) and tap water. All experimental procedures were conducted in the morning period (7–12 A.M.) and were in accordance with the Ethical Committee for Animal Use of the School of Medicine of Ribeirao Preto, University of Sao Paulo (protocol 185/2010).

### Experimental design

**Figure 1:**
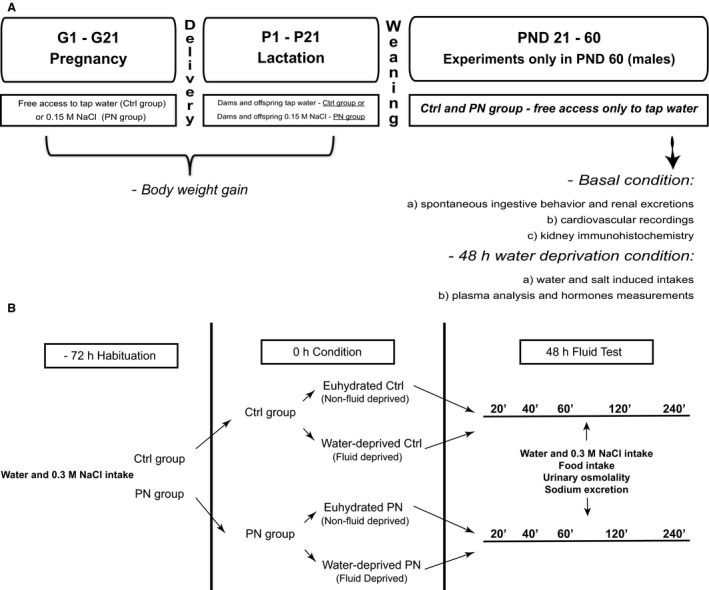
Schematic diagram showing experimental design and protocols.

Rats were mated and housed in collective plastic cages (one couple per cage). After mating, verified by the presence of copulatory plug, male rats were removed and the pregnant female rats were maintained in individual cages. Then, dams were assigned into two groups and given either water (Ctrl) or 0.15 mol/L NaCl (perinatal 0.15 mol/L NaCl: PN) during the entire pregnancy‐lactation period. Within 24 h after birth, litter size was adjusted to eight pups, retaining the same number of males and females in each litter, to avoid changes in maternal behavior (Moore and Morelli 1979; Alleva et al. 1989; Hao et al. 2011). Dams continued to receive their respective perinatal treatment (water or 0.15 mol/L NaCl) until pups were weaned at postnatal day 21. At weaning, males of both experimental conditions were kept in standard conditions of water and food until postnatal day 60 (PND 60), when all the experiments were performed (Fig. 1). No more than 3 male adult rats from the same dam were used for the same condition in each experiment. We analyzed only the male rats because changing estrogen levels can influence the female behavior and other neuroendocrine systems (Stricker et al. 1991; Chow et al. 1992; Dalmasso et al. 2011; Vilhena‐Franco et al. 2011).

### Experimental protocols

#### Basal conditions

##### Experiment #1a: Body weight gain

Dams from Ctrl and PN group were weighed in the beginning and end of the pregnancy and every week during lactation. Both groups of litters were weighed every week during the lactation period and after the weaning, until PND 60.

##### Experiment #1b: Spontaneous ingestive behavior and renal excretion

PND 60 male rats were placed in individual metabolic cages with access to commercial diet (@Quimtia, Buenos Aires, Argentina), tap water and hypertonic (0.3 mol/L) NaCl solution in graduated bottles. Daily spontaneous fluid intake, food ingestion, urinary osmolality and Na^+^ excretion were recorded.

##### Experiment #1c: Cardiovascular recordings

A separate group of adult male offspring was used for the determination of mean arterial pressure (MAP) and heart rate (HR) by femoral artery cannulation, at PND 60.

##### Experiment #1d: Kidney immunohistochemistry

An additional set of adult offspring (Ctrl and PN) (different from experiments #1b and #1c) was decapitated at PND 60 and the kidney was removed to determine the number of ED1‐positive cells by immunohistochemistry. ED1, a cellular antigen found in activated macrophages and monocytes, was monitored as a surrogate indicator of the development of an inflammatory response.

#### Water deprivation challenge

##### Experiment #2a. Water and salt induced intakes

Ctrl and PN adult male rats were individually housed in metabolic cages for 3 days with free access to standard food, 0.3 mol/L NaCl and tap water for habituation. After this period, animals were randomly divided in two groups: one remained in the same conditions as in the habituation period (euhydrated) and another was maintained for the next 48 h with free access to standard chow but no access to fluids (water‐deprived). Therefore, 4 groups were originated: Ctrl euhydrated, Ctrl water‐deprived, PN euhydrated and PN water‐deprived.

On the morning of the experimental day, graduated bottles containing water and 0.3 mol/L NaCl were returned to the cages. Water, 0.3 mol/L NaCl and food intakes, as well as urine osmolality and sodium excretion, were evaluated in 20 min intervals during 240 min. Salt preference ratio was also calculated: (0.3 mol/l NaCl intake/water + 0.3 mol/L NaCl intakes) × 100 (Frankmann and Dorsa 1986). Finally, total NaCl ingestion (mmol/L) was calculated as [(0.3 mol/L NaCl intake (mL/100 g bw) × molality of the hypertonic sodium solution (450 mmol/L NaCl))/total fluid intake (mL/100 g bw)]. (Fig. 1B).

##### Experiment #2b: Plasma analysis and hormones measurements

Ctrl and PN adult male rats, submitted or not to water deprivation, were euthanized by decapitation and trunk blood was collected into iced cold polypropylene tubes for the determination of plasma osmolality and concentrations of Na^+^, protein, vasopressin (AVP), oxytocin (OT), atrial natriuretic peptide (ANP), ANGII and corticosterone, or into heparinized capillaries for the determination of hematocrit.

### Cardiovascular recordings

For cardiovascular measurements, animals were anesthetized with an intraperitonial injection of ketamine (55 mg/kg) plus xylazine (10 mg/kg) and a polyethylene catheter was inserted into the left femoral artery. The opposite end of the catheter was tunneled back underneath the skin and exposed between the scapulae. After the surgical procedure, all rats received analgesic and antibiotic drugs (carprofen, 5 mg/kg subcutaneously, and gentamicin 25 mg/kg intramuscular). Twenty‐four hours after, the arterial catheter was connected to a pressure transducer (MLT844, AD Instruments, Sydney, Australia) and coupled to an acquisition system (Power Lab ML866/P, AD Instruments, Sydney, Australia). Mean arterial pressure (MAP) and heart rate (HR) were derived from the arterial pulse pressure as maximum, minimum, integral of the arterial pulse pressure wave, and the inverse of inter‐beat interval of the arterial pulse pressure wave, respectively.

### Imunohistochemical kidney analysis

Animals were decapitated and the kidneys were removed, sectioned, and fixed in metacarn solution (60% v/v methanol, 30% v/v chloroform and 10% v/v acetic acid) for 24 h, followed by washes in 70% v/v alcohol and embedding in paraffin. Tissue blocks from all the experimental groups were cut in a microtome (8 *μ*m) and placed in three consecutive washes of xylene to remove paraffin. Then, they were placed in three washes of alcohol in decreasing concentrations for the hydration of tissues. The slides were incubated for 10 min in a solution containing 10% w/v sodium azide and 30% v/v hydrogen peroxide to block the endogenous peroxidase activity. Afterwards, the sections were incubated overnight at room temperature with primary antibody anti‐ED1 (1:1000, produced in mouse, Serotec, UK), which reacts with a cytoplasmatic antigen present in macrophages and monocytes. Then, the sections were incubated with a secondary biotinylated antibody produced in donkey (1/200, Vector Laboratories) for 30 min, followed by another 30 min of incubation in a solution containing avidin‐biotin (Vector Laboratories). The color was developed by adding DAB chromogen (Sigma) and 1% nickel chloride in the presence of H_2_O_2_. Counterstaining was performed with methylgreen. The number of ED1 (macrophages/monocytes)‐positive cells in glomeruli and renal cortical tubulointerstitium were counted through the examination of 50 glomeruli and 30 grid fields (measuring 0,100 mm^2^), respectively, and the mean counts per kidney were calculated.

### Determination of hematocrit and plasma sodium, osmolality and protein concentrations

Plasma Na^+^ concentrations were determined using a flame photometer (Micronal, B262). Plasma osmolality was determined by the method of lowering the freezing point of water by means of a micro‐osmometer (Precision Systems, Inc.). Values obtained were expressed in mOsm/kg H_2_O. To determine hematocrit, trunk blood was collected into a heparinized capillary, which had one extremity occluded. Capillaries were centrifuged at 106 *g* for 5 min and then read against an appropriate volumetric scale and Hct expressed as percentage of red blood cells. For the determination of plasma protein concentrations, animals were euthanized by decapitation. Trunk blood was collected into ice‐cold tubes containing 10 *μ*L of heparin per mL of blood. Plasma was separated by centrifugation at 1008 *g* at 4°C for 20 min, and then stored at −20°C. Aliquots of 10 *μ*L of plasma were diluted at 1:200 and processed for the analysis of protein content by the Bradford method (Bradford 1976).

### Determination of plasma OT, AVP, ANP, ANGII, and corticosterone concentrations

Plasma concentrations of AVP, OT, ANP, ANGII, and corticosterone were measured by specific radioimmunoassay as previously described (Gutkowska et al. 1984; Botelho et al. 1994; Haanwinckel et al. 1995; Elias et al. 1997; Durlo et al. 2004). AVP and OT were extracted from 1 mL of plasma with acetone and petroleum ether, while corticosterone was extracted from 25 *μ*L of plasma with 1 mL of ethanol. ANP and ANG II were extracted from 1 mL of plasma using Sep‐Pak C‐18 cartridges (Waters Corporation, Milford, MA). Assay sensitivity and intra‐ and inter‐assay coefficients of variation were 0.12 pg/mL, 4.9% and 4.3% for AVP; 0.12 pg/mL, 1.92% and 18.3% for OT; 0.75 pg/mL, 3.8% and 19.2% for ANP; 0.39 pg/mL, 4.17% and 10.3% for ANG II and 0.12 *μ*g/dL, 3.70% and 8.2% for corticosterone.

### Statistical analysis

The results are presented as means ± standard error (SEM). Data were analyzed using Statistica software (StatSoft, Tulsa, OK). Statistical differences between two experimental groups (in the case of basal behavioral assessments, cardiovascular parameters and kidney ED‐1 expression) were evaluated using independent sample unpaired Student's two‐tailed *t* tests. For water deprivation analyses, Two‐way ANOVA (with or without repeated measures) and *Bonferroni* multiple‐comparisons post‐hoc test were used, considering the intakes (water and 0.3 mol/L NaCl), plasma sodium, osmolality and protein, hematocrit and hormone concentrations as dependent variables and the programming effect (Ctrl and PN) and hydration status (euhydrated and water‐deprived) as the independent variables. Post‐hoc comparisons were only performed when the *F*‐value showed a significant effect and/or interaction. The level of significance was set at 5%.

## Results

### Basal conditions

#### Experiment #1a. Body weight gain

**Figure 2:**
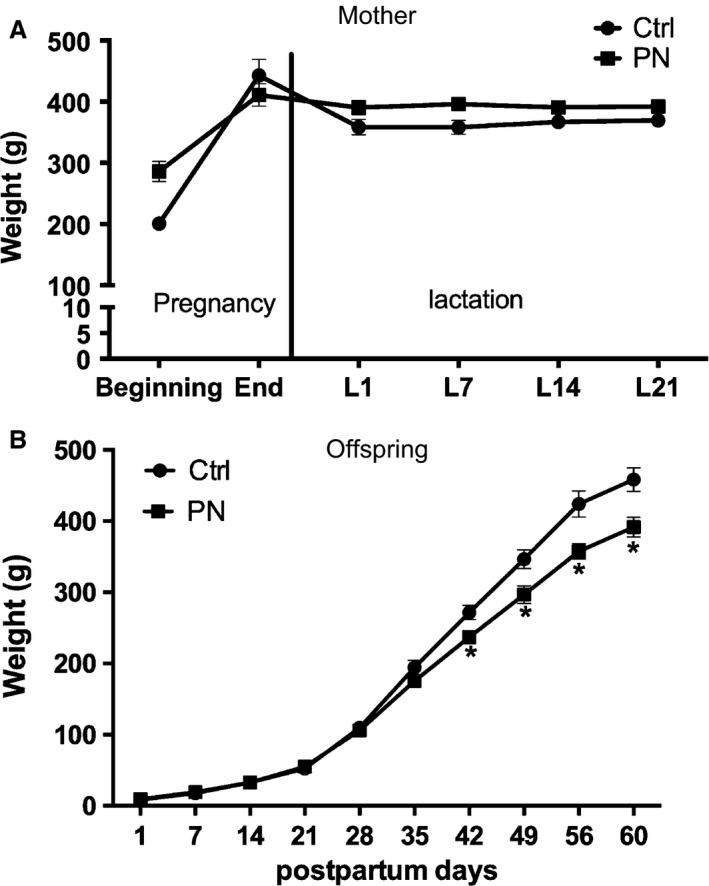
Weight gain (g) of mothers (A) and male adult offspring (B) of Ctrl and PN groups. Results are presented as mean ± SEM. *P < 0.05 versus Ctrl. *N* = 6–7.

Our results showed that exposure to 0.15 mol/L (isotonic) NaCl solution did not change maternal body weight during the pregnancy‐lactation period (Fig. 2A). In PN male offspring, body weight was significantly lower only after PND 42, when compared with Ctrl group (*F*_1,145_ = 37.9, *P* < 0.05) (Fig. 2B).

#### Experiment #1b. Spontaneous ingestive behavior and renal excretion

**Figure 3:**
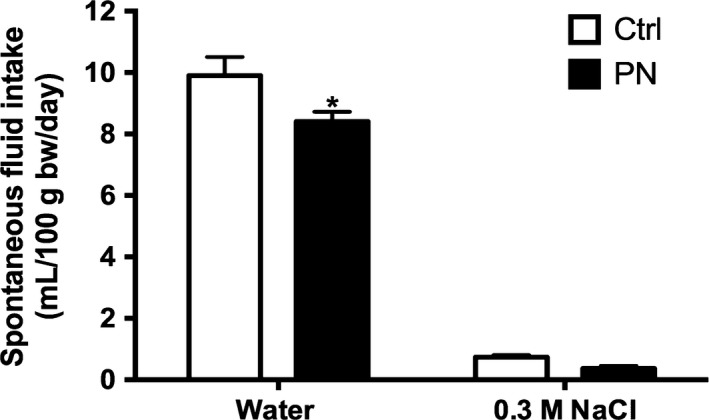
Spontaneous water and salt intake cumulative intakes (mL/100 g bw/day) male adult offspring (B) of Ctrl and PN groups. Results are presented as mean ± SEM. *P < 0.05 versus Ctrl. *N* = 8–12.

Figure 3 shows that the mean daily spontaneous water intake was diminished in PN animals (*T*_16_ = 2.315, *P* < 0.05) when compared with Ctrl group, whereas daily 0.3 mol/L NaCl intake was not altered. Furthermore, PN offspring exhibited increased food intake (*T*_16_ = 2.781, *P* < 0.05), increased urinary volume (*T*_13_ = 7.267, *P* < 0.01), increased urinary sodium excretion (*T*_14_ = 3.762, *P* < 0.01) and unchanged urinary osmolality (Table 1).

**Table 1:** Food intake, urinary osmolality, urinary sodium, and urinary volume measured in Ctrl and PN male adult offspring (PND 60)

Groups	Food intake (g/100 g bw/24 h)	Urinary osmolality (mOsm/Kg H_2_O/24 h)	UNa^+^.V (*μ*Eq/100 g bw/24 h)	Urinary volume (mL/100 g bw/24 h)
Ctrl	7.220 ± 0.31 (10)	1436 ± 287.2 (18)	40.05 ± 5.853 (8)	0.95 ± 0.10 (6)
PN	8.390 ± 0.39 (10)^a^	1403 ± 253.6 (15)	73.35 ± 6.641 (8)^a^	2.989 ± 0.21 (9)^a^

Values are means ± SEM, (*n*).

a *P* < 0.05 versus Ctrl.

#### Experiment #1c. Cardiovascular recordings

Our results showed that the perinatal 0.15 mol/L NaCl intake did not induce any significant changes in the MAP or HR of adult male offspring (Table 2).

**Table 2:** Mean arterial pressure (MAP), heart rate (HR) of male adult Ctrl and PN offspring (PND 60)

Groups	MAP (mmHg)	HR (beats min^−1^)
Ctrl	97.22 ± 3.036 (9)	322.6 ± 9.060 (9)
PN	100.3 ± 1.54 (10)	336.1 ± 9.808 (10)

Values are means ± SEM, (*n*). HR, heart rate

#### Experiment #1d Kidney immunohistochemistry

**Figure 4:**
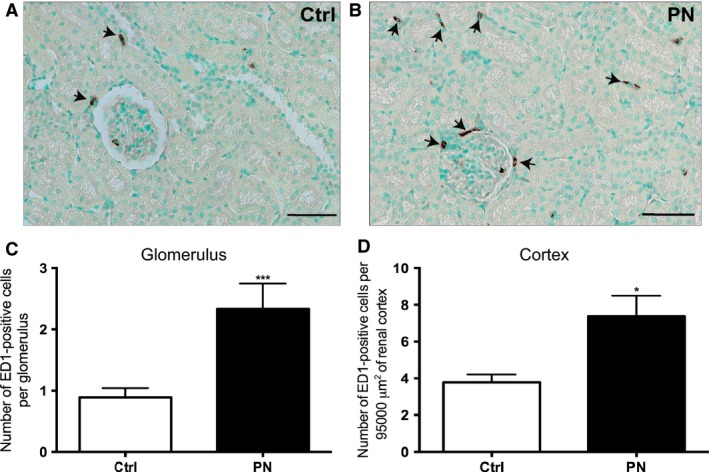
(A) Representative sections showing the imunohistochemical localization of ED‐1 (macrophages/monocytes) positive cells (arrow) in renal cortex and (B) number of ED1‐positive cells per glomerulus or per area of renal cortical tubulointerstitium of Ctrl and PN offspring. Counterstaining with methylgreen. Magnification: 280x. Scale bar: 50 *μ*m. Results are presented as mean ± SEM. *^**^ *P* < 0.01 or * *P* < 0.05 versus Ctrl. *N* = 7–9.

Programming effects were observed in the kidney of adult offspring exposed to 0.15 mol/L NaCl during pregnancy and lactation, since a higher number of ED‐1 positive cells (macrophages/monocytes) were found in glomeruli (*T*_14_ = 2.969, *P* < 0.01) (Fig. 4C) and tubulointerstitium (*T*_14_ = 2.698, *P* < 0.05) (Fig. 4D) of PN renal cortices, indicating a significant inflammatory state in these areas (Fig. 4A and B).

### Water and salt intake after 48‐h water deprivation

#### Experiment #2a Water and salt intake

**Figure 5:**
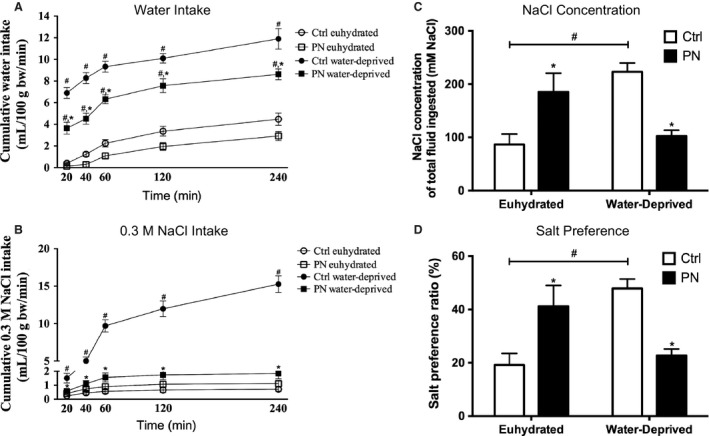
Cumulative water (A) and 0.3 mol/L NaCl (B) intakes in Ctrl and PN male adult offspring subject or not to 48‐h water deprivation. (C) Sodium chloride concentration of total fluid (mmol/L NaCl) and (D) salt preference (Argüelles et al. 1996). Open circle: Ctrl euhydrated; open square: PN euhydrated; filled circle: Ctrl water‐deprived; filled square: PN water‐deprived. Results are presented as mean ± SEM. *P < 0.05 versus Ctrl euhydrated or water‐deprived and ^#^ *P* < 0.05 versus euhydrated groups. *N* = 6–10.

As expected, 48 h of water deprivation increased cumulative water (*F*_1,29_ = 248.3, *P* < 0.001) and 0.3 mol/L NaCl (*F*_1,29_ = 216.8, *P* < 0.001) intakes, whereas decreased food ingestion (*F*_1,29_ = 238.4, *P* < 0.001) in Ctrl group. These effects occurred in parallel with an increased urinary osmolality (*F*_1,29_ = 92.01, *P* < 0.0001) and sodium concentrations (*F*_1,25_ = 123.5, *P* < 0.0001), when compared with Ctrl euhydrated group (Table 3). Interestingly, PN water‐deprived rats drank less water (*F*_1,29_ = 31.91, *P* < 0.001) (Fig. 5A) and 0.3 mol/L NaCl (*F*_1,29_ = 145.3, *P* < 0.001), when compared with the Ctrl water‐deprived group (Fig. 5B). Our results showed that sodium chloride concentration of the total fluid ingested (mmol/L NaCl) (*F*_1,25_ = 15.06 *P* < 0.05) and salt preference (%) (*F*_1,26_ = 15.02, *P* < 0.05) of the male adult offspring was significantly altered by maternal sodium chloride ingestion. The PN male adult offspring, in a basal condition, showed an increase in sodium chloride concentration of the total fluid ingested and salt preference compared with Ctrl euhydrated. After 48 h of water deprivation, the PN animals showed a decrease in those both parameters analyzed compared with Ctrl water‐deprived (Fig. 5C and D).

**Table 3:** Food intake, urinary osmolality and urinary sodium measured in male adult euhydrated or 48‐h water‐deprived Ctrl and PN offspring (PND60)

Groups	Food intake (g/100 g bw/4 h)	Urinary osmolality (mOsm/Kg H_2_O/4 h)	UNa^+^.V (*μ*Eq/100 g bw/4 h)
Ctrl Euhydrated	7.40 ± 0.34 (8)	473.94 ± 148.30 (10)	46.46 ± 11.56 (8)
PN Euhydrated	8.39 ± 0.38 (10)	849.89 ± 205.90 (10)	66.34 ± 11.20 (8)
Ctrl Water‐Deprived	3.08 ± 0.20 (6)^a^	2637.61 ± 209.47 (8)^a^	286.97 ± 34.51 (8)^#^
PN Water‐Deprived	2.94 ± 0.16 (9)^a^	2508.51 ± 161.03 (5)^a^	341.58 ± 24.90 (5)^a^

Values are means ± SEM, (*n*).

a *P* < 0.05 versus euhydrated groups respectively.

#### Experiment #2b Plasma analysis and hormones measurements

**Figure 6:**
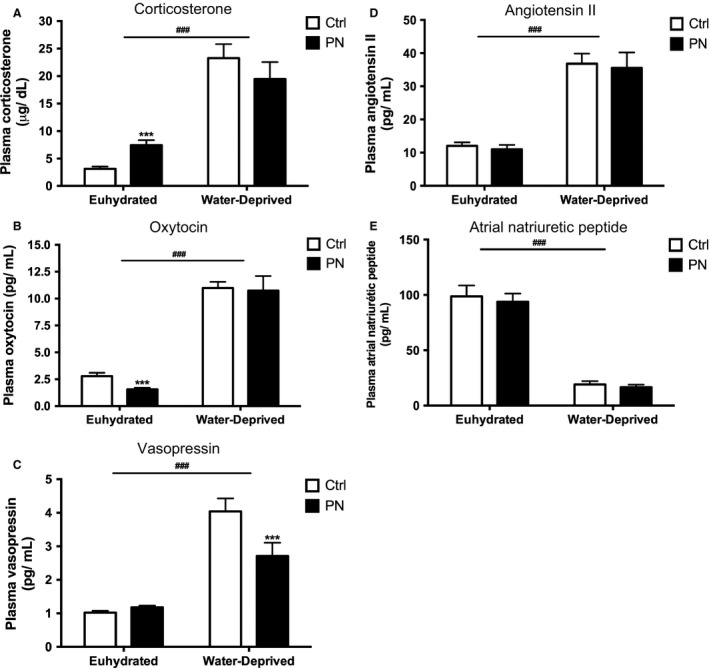
Corticosterone (A), oxytocin (B), vasopressin (C), angiotensin II (D) and atrial natriuretic peptide (E) plasma concentrations in euhydrated and water‐deprived adult offspring of Ctrl and PN groups. Results are presented as mean ± SEM. *^**^ *P* < 0.001 versus Ctrl water‐deprived and ^###^ *P* < 0.001 versus euhydrated groups. *N* = 9–51.

As expected, water deprivation induced a significant increase in hematocrit (*F*_1,30_ = 168.2, *P* < 0.001), plasma osmolality (*F*_1,34_ = 127, *P* < 0.001), plasma sodium (*F*_1,35_ = 28.71, *P* < 0.001) and plasma protein concentrations (*F*_1,41_ = 50.09, *P* < 0.001) (Table 4), being these effects independent of 0.15 mol/L NaCl exposure. Water‐deprived animals also exhibited increased plasma levels of AVP (*F*_1,109_ = 196.4, *P* < 0.001), OT (*F*_196_ = 357.3, *P* < 0.001), ANGII (*F*_1, 62_ = 134.5, *P* < 0.001) and corticosterone (*F*_1,58_ = 115.1, *P* < 0.001), and decreased ANP plasma levels (*F*_1,90_ = 41.62, *P* < 0.001). Conversely, PN euhydrated animals showed increased plasma corticosterone (*P < 0.05*; Fig. 6A) and decreased plasma OT concentrations (*P *< 0.05; Fig. 6B), when compared with the respective Ctrl group. AVP, ANGII, and ANP plasma concentrations showed no significant differences between euhydrated PN and Ctrl groups (Figs 6C, D, and E, respectively). Our results also showed that PN water deprivation rats reduced AVP secretion (*F*_1,109_ *= *13.12*, P < 0.001*) in comparison with Ctrl animals. No changes were observed in OT, ANP, ANGII, and corticosterone plasma concentrations between PN and Ctrl water‐deprived animals (Fig. 6).

**Table 4:** Hematocrit, plasma osmolality, sodium and protein concentrations in male adult euhydrated or 48‐h water‐deprived Ctrl and PN offspring (PND60)

Groups	Hematocrit (%)	Plasma osmolality (mOsm/Kg H_2_O)	Plasma sodium (mEq/L)	Plasma protein (g/dL)
Ctrl Euhydrated	42.90 ± 1.36 (21)	296.2 ± 2.83 (19)	139.81 ± 1.48 (11)	6.31 ± 0.27 (24)
PN Euhydrated	45.00 ± 1.04 (19)	300.8 ± 2.09 (15)	138.12 ± 0.66 (8)	6.49 ± 0.18 (36)
Ctrl Water‐Deprived	54 ± 0.78 (10)^a^	313.81 ± 2.62 (11)^a^	148.5 ± 1.42 (12)^a^	9.11 ± 0.61 (6)^a^
PN Water‐Deprived	55.12 ± 1.04 (8)^b^	316.55 ± 1.86 (9)^b^	144.87 ± 1.61 (8)^b^	9.15 ± 0.21 (9)^b^

Values are means ± SEM, (*n*).

a *P* < 0.05 versus Ctrl euhydrated.

b *P* < 0.05 versus PN euhydrated.

## Discussion

The present study investigated the hypothesis that maternal and neonatal exposure to mild but chronic salt ingestion may significantly affect the offspring, determining changes in homeostatic regulation. First, we confirmed that exposure to 0.15 mol/L NaCl solution does not affect the maternal weight gain, which is consistent with the previous findings showing that pregnant dams efficiently increase their urinary flow in order to manage the ECF volume, and therefore, maintain body weight (Deloof et al. 2000). Regarding the impact of this challenge to offspring, epidemiological studies have demonstrated that different kinds of stress during intrauterine development increase the incidence of cardiovascular and metabolic diseases in adulthood (Barker 1986, 1995). Accordingly, we showed a significant decrease in weight of young PN animals, which began at PND 42 and persisted until adult life (60 days old). At this time point, PN animals also exhibited increased food intake (despite the decreased body weight) and increased urinary volume and sodium excretion, indicating that, besides affecting hydroelectrolytic homeostasis, exposure to salt during the ontogenic period induces important changes in energy homeostasis (Table 2) (weight loss and polyphagia) (Coelho et al. 2006; Nicolaïdis 2008; Reynolds et al. 2014; World Health Organization 2015).

However, no changes in urinary osmolality, hematocrit, plasma protein concentration, plasma osmolality, MAP, and HR were observed in PN animals, indicating that their osmoregulatory systems were probably subjected to an imprinting process, which allowed them to be adapted to a new allostatic situation in terms of body fluid homeostasis and blood pressure regulation. These findings are in agreement with Curtis et al. (2004), who showed similar results in offspring originated from mothers fed with a high‐salt diet during pregnancy. However, PN animals exhibited a moderate kidney inflammation, characterized by an increased number in ED‐1 positive cells (monocytes and macrophages) in the vascular and tubular portions of the nephron. These results suggest that increased salt exposure induces not only functional but also structural changes in important effector organs regulating hydromineral balance, such as the kidneys. These data are in agreement with the reports of Koleganova et al. (2011), who verified that maternal high‐salt intake leads to reduced final number of glomeruli and albuminuria in the offspring.

Previous studies have reported that early challenges lead to persistent changes in both spontaneous and stimulated fluid intake in adult animals (Nicolaidis et al. 1990; Argüelles et al. 2000; Curtis et al. 2004; Macchione et al. 2012). However, those results remain controversial, particularly with regard to the effect (i.e. increase or decrease) produced by perinatal manipulation on fluid ingestion and mechanisms underlying these alterations. Previous works (Macchione et al. 2012; Macchione et al. 2015) showed that spontaneous hypertonic NaCl intake during pregnancy and lactation reduces water intake in adult offspring in response to furosemide‐induced sodium depletion, with no changes in saline intake. According to these authors, more important than the total amount of ingested fluids was the proportion between them, which allowed these programmed animals to consume, as an anticipatory mechanism, a more hypertonic mixture.

Similarly, our results showed that PN descendants, when compared with Ctrl animals, drank less water and an unaltered volume of 0.3 mol/L NaCl under basal conditions, which also resulted in a relative hypertonic load, and consequently, an increased Na^+^ preference ratio. In response to water deprivation, however, both water and 0.3 mol/L NaCl intakes were reduced in PN animals, producing a decrease in Na^+^ preference ratio and total NaCl ingested, when compared with the respective PN euhydrated group. This inability of PN animals to acquire (drink) or retain (reabsorb) water in response to water deprivation probably reflected the lower AVP secretion found in this group, resulting in increased plasma osmolality, Na^+^ and protein concentrations. These results are consistent not only with the renal antidiuretic effect of AVP, but also with its central role as a dipsogenic peptide (Szczepanska‐Sadowska et al. 1987) and support the hypothesis that the sensitization of osmoregulatory systems by a prolonged NaCl exposure may lead to an increased threshold for the induction of thirst and blunted hormone release in face of a new challenge (Digby et al. 2010).

Importantly, we also measured the circulating plasma levels of other hormones that are responsive to hydromineral balance and blood pressure regulation. First, we saw no changes in the basal and water deprivation‐induced release of ANGII and ANP. This is not surprising from the cardiovascular perspective, given the fact that the effective circulatory volume (ECV) is the primary regulator of the secretion of these peptides (Gutkowska et al. 1984; Antunes‐Rodrigues et al. 2004) and we have provided indirect evidence, by means of total plasma protein concentrations, that ECV is not altered in PN animals compared with Ctrl, either under basal or stimulated conditions. However, ANP normally counteracts the dipsogenic and natriorexigenic central roles of ANGII (Galli and Phillips 1996), suggesting that these systems may be also altered by programming in PN animals, since fluid intake behavior was altered in basal conditions and also in response to water deprivation, despite the unchanged ANP/ANGII plasma levels found in this group.

On the other hand, besides ANP, OT secretion responds to elevations in plasma osmolality or volume by centrally limiting sodium appetite and increasing sodium excretion (Blackburn et al. 1993). Both peptides exhibit a natriuretic action, an important mechanism underlying the creation of an osmotic gradient that favors renal water excretion and thereby reestablishes ECF volume. Despite the unaltered ANP levels, we detected an important decrease in OT secretion in PN descendants under basal conditions. This is surprising, considering that PN animals exhibited an increased basal Na^+^ urinary excretion, suggesting that other local, neural, or endocrine factors regulating Na^+^ balance are still operating in this experimental situation. In response to water deprivation, however, OT secretion was maintained, differently from AVP, as we had previously discussed in this section. These results contrast with those reported by McDonald et al. (1998), who demonstrated that vasopressinergic cells of sheep fetal hypothalami were less responsive than oxytocinergic neurons to an acute osmotic challenge (intravenous 20% w/v mannitol) performed to dams.

In addition to parturition, lactation, and natriuresis, OT has emerged as a core component of the mechanisms mediating health benefits and antistress effects, such as increased anabolic metabolism and, in some circumstances, growth, and behavioral calmness (Witt et al. 1992; Uvnäs‐Moberg 1998; Heinrichs et al. 2003). Central OT has been described as an important regulator of the stress response and is believed to attenuate the response of the hypothalamic‐pituitary‐adrenal (HPA) axis (Windle et al. 1998; Neumann et al. 2000a,b). Exposure to stressful environments induces corticotrophin‐releasing hormone (CRH) release from neurons located at the hypothalamus and drives adrenocorticotrophic hormone (ACTH) release from the anterior pituitary, which in turn prompts corticosterone release from the cortex of adrenal glands (Ulrich‐Lai and Herman 2009). The mechanism of OT‐induced HPA axis suppression is mediated by cAMP‐responsive element‐binding protein (CREB), which not only controls the activity of CREB‐regulated transcriptional cofactors (CRTC2/3), but also transcription of CRH in the hypothalamus (Liu et al. 2011; Aguilera and Liu 2012; Jurek et al. 2015; Silberman et al. 2015). Phosphorylated CRTCs are sequestered in the cytoplasm, but rapidly dephosphorylated and translocated into the nucleus following a stressful stimulus.

It has been proposed by these studies that OT release in response to stress operates sequestering CRTC3 in the cytosol, resulting in its reduced binding to CRH gene promoter, and ultimately, decreased CRH gene expression. Dexamethasone‐induced Ras‐related protein 1 (RASD1), a small G‐protein, is upregulated in the hypothalamus following an increase in plasma osmolality and possibly constitutes a parallel pathway through which CREB activity can be inhibited in response to osmotic stress (Greenwood et al. 2016). These findings are in agreement with our results, since maternal exposure to 0.15 mol/L NaCl reduced basal OT release in adult male offspring, being this effect accompanied by an increase in the activity of the HPA axis, assessed in the present study by an increase in corticosterone plasma levels. Corticosterone is released in a circadian and ultradian fashion (Lightman et al. 2002; Joëls et al. 2012) as well as due to HPA axis activation in response to stressors such as increased extracellular volume and osmolality (Durlo et al. 2004; de Kloet et al. 2005; Ruginsk et al. 2007; Baram and Joels 2009). It has been demonstrated that an adverse early environment is associated with enhanced autonomic and HPA responses to stress during adulthood, what may predispose adult individuals to an increased disease risk (van Praag et al. 2000; Phillips and Jones 2006; Maccari et al. 2014; Marco et al. 2014).

Future studies will be required to establish whether sex differences exist in the effect of perinatal programming on hydromineral balance. Additionally, it will be important to determine if the programming event is exposure period‐dependent one. In other words, does the programming event occur during gestation or lactation alone or does it require both exposure periods and if changes in adrenal responses to sodium challenge exist (e.g. aldosterone production and release)? Additionally, it would be important to determine if the exposure to 0.15 mol/L NaCl during lactation changes the composition of the milk. Finally, would perinatal programing alter the responsiveness of compensatory responses to a non‐osmotic challenge such as hypovolemia?

In summary, the present results confirm that the intrauterine programming can occur at any stage of fetal and/or postnatal development and may affect not only the physiology of organs and systems, but also function (Bateson et al. 2004; Ross and Desai 2005). These changes can be isolated or scattered events, with poor or notable results in the animal development, depending on the type of insult (Fowden et al. 2006). Therefore, the switched pattern of water to salt intake could be considered a major environmental stressor that, experienced early in life, significantly affects key regulatory mechanisms of water balance maintenance in adult offspring, like reduced water intake and AVP release in response to a water deprivation challenge. Furthermore, the progeny showed endocrine and renal stress signs that may contribute to the development of other comorbidities. Our data also showed that plasma levels of corticosterone were significantly higher in PN offspring. From an evolutionary perspective, changes in the pattern of HPA axis activation are necessary because they allow systems to respond promptly to temporary or long‐lasting physiological and pathological conditions. This plasticity allows individuals exposed to unfavorable environmental conditions to fine‐tune their response for environmental challenge later in life.

## Conflict of Interests

The authors declare that they have no competing interests.
